# Successful management of rare gingival metastasis from gastric adenocarcinoma: a case report and literature review

**DOI:** 10.1186/s12957-017-1215-3

**Published:** 2017-08-01

**Authors:** Zhili Wu, Jun Tang, Yan Li, Husheng Lu, Jun Xu, Donglai Lv

**Affiliations:** 1 Department of Clinical Oncology, The 105 Hospital of People’s Liberation Army, 105 Hospital of PLA, Hefei, Anhui, 230031 China; 2 Department of Gastroenterology and Hepatology, The 105 Hospital of People’s Liberation Army, Hefei, Anhui China; 3 Department of Pathology, The 105 Hospital of People’s Liberation Army, Hefei, Anhui China

**Keywords:** Gastric carcinoma, Gingiva, Neoplasm metastasis

## Abstract

**Background:**

Gastric cancer rarely metastasizes to the oral cavity, especially to gingiva. Only 18 cases have been reported worldwide to date. This paper herein presents the nineteenth case of gingival metastasis from gastric cancer.

**Case presentation:**

A 75-year-old man who underwent a radical gastrectomy for gastric adenocarcinoma was admitted to clinical oncology center for gingival mass which was originally diagnosed as epulis. The subsequent positron emission tomography-computed tomography (PET-CT) and histopathological examination revealed a gingival metastatic adenocarcinoma originated from gastric carcinoma. Then three-dimensional conformal radiotherapy (3D–CRT) with synchronization and sequential chemotherapy demonstrated clinical benefit in this patient. Furthermore, this research reviewed the records of 18 cases of gingival metastasis from gastric carcinoma in English, Japanese, and Chinese literature, and summarized the clinicopathologic features of the disease based on previously published papers.

**Conclusion:**

This case suggests that gingival metastasis from gastric cancer is worthy of vigilance. Biopsy and immunohistochemical (IHC) staining should be used for the final diagnosis. Moreover, the patient with uncommon gingival metastatic lesion can be successfully treated by radiotherapy with adjuvant chemotherapy.

## Background

Metastatic neoplasia of the oral cavity is extremely rare, accounting for approximately 1% of all malignant oral tumors [1]. Earlier publications reporting on metastatic oral malignancy covering periods over recent decades elucidated that the breast, lung, kidney, and colon are places where primary malignancy is widely found, which make up about 70% of all cases [2]. However, the stomach comprises only 1.8% of all origin discovered in the oral cavity metastasis [3], with only a few sporadic cases reported in the literature. Because of the rare incidence, accurate diagnosis of this metastasis is enough of a challenge. The final diagnosis of gingival metastasis usually predominantly depends on pathological characteristics, and no effective treatments have been reported. This paper reported a male patient who presented with a gingival lump that was determined to be a metastasis from a primary gastric adenocarcinoma and found that the combination of radiochemotherapy led to improved clinical benefit.

## Case presentation

**Fig. 1:**
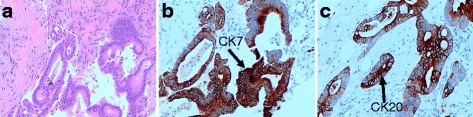
Histopathological examination of primary gastric carcinoma. **a** The clusters of hyperchromatic and pleomorphic cancer cells were observed under optical microscope. The primary tumor was composed of poorly differentiated adenocarcinoma cells. (hematoxylin and eosin staining, ×100). The IHC investigations of primary tumor specimens demonstrated immunoreactivity of CK7 (**b**) and CK20 (**c**). (Diamino-benzidine staining, ×100)

A 75-year-old Chinese male, who was diagnosed with gastric adenocarcinoma and who underwent a radical total gastrectomy, was admitted to the clinical oncology center on 27 June 2016 due to the presence of an indolent lump on his gingiva. The patient complained a gingival swelling that continued for approximately 1 month with progressive enlargement. A physical examination revealed a gray and painless neoplasm on the left upper gingiva with local hemorrhage on palpation. The diameter of mass was 2 cm. His dentist had originally diagnosed epulis and suggested surgical excision. However, the patient refused. He was treated with antibiotics and non-steroidal anti-inflammatory drugs, but the result was negative. Examination of the patient’s medical history revealed that the patient underwent a radical gastrectomy through laparoscope in 29 months earlier. The histological examination of the postsurgical specimen confirmed that the muscular layer of gastric wall had been infiltrated by poorly differentiated adenocarcinoma cells, and lymph nodes metastasis (2/18) was also shown. The clusters of hyperchromatic and pleomorphic cancer cells among the tumor tissue were observed under optical microscope (Fig. 1a). Furthermore, immunohistochemical (IHC) staining showed that tumor was positive for CK7 (Fig. 1b) and CK20 (Fig. 1c), and negative for Her-2, CK-19, and TTF1. There was no cancerous cell in the greater omentum, in the proximal resection margin or in the distal resection margin according to the histopathology examination, and no distant metastasis was found by a computerized tomography (CT) scan. The stage of gastric cancer was pT3N1M0 (stage IIIA). The patient received 4 cycles of chemotherapy (oxaliplatin 130 mg/m^2^ in day 1 and tegafur 800 mg/m^2^ in days 1–4, this regimen was performed once every 4 weeks as 1 cycle) after the operation. After 2 months of chemotherapy, the contrast-enhanced CT scan showed no recurrence of the tumor. No regular check-up and followed-up were performed.

**Fig. 2:**
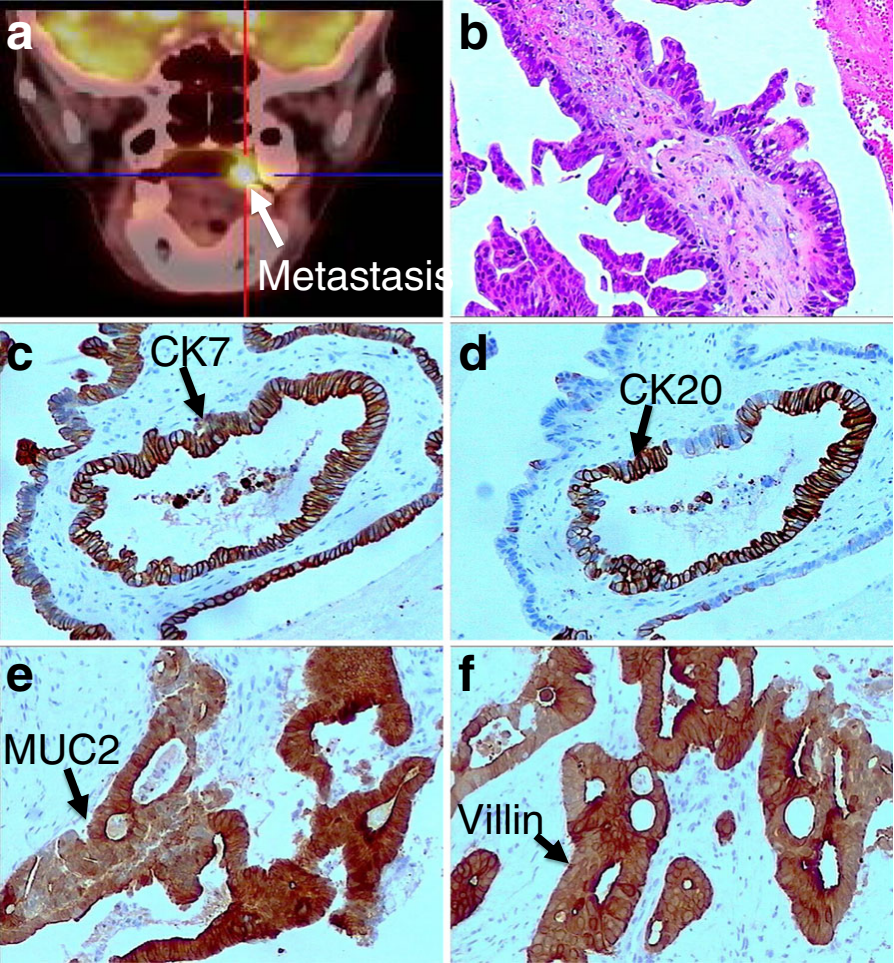
PET-CT and histopathological analysis of the gingival neoplasm. **a** PET-CT showed hypermetabolic foci of the upper gingiva (*left molar area*), which were considered the malignant lesions. **b** The section of biospy showed metastatic foci of a poorly differentiated adenocarcinoma cells. (hematoxylin and eosin staining, ×100). **c**–**f** The IHC investigations of gingival tumor specimens demonstrated immunoreactivity of CK7, CK20, MUC2, and Villin. (Diamino-benzidine staining, ×100)

At admission, serum level of carcinoembryonic antigen (CEA) was elevated (13.12 ng/ml). On 30 June 2016, a PET-CT revealed left adrenal gland and upper gingiva (left molar area) involvement, which were considered as the malignant lesions (Fig. 2a). The gingival mass was subsequently getting a surgical biopsy. Samples were fixed in 10% neutral formaldehyde and embedded in paraffin. Serial sections were cut at a thickness of 10 μm. The results showed that tumor cells were arranged in pseudoglandular patterns and were uniform in size with prominent nuclei microscopically (Fig. 2b). The IHC investigations of gingival tumor postoperative specimens demonstrated immunoreactivity of CK7 (scattered+) (Fig. 2c), CK20 (partly+) (Fig. 2d), MUC2 (+) (Fig. 2e), Villin (+) (Fig. 2f), NapsinA (−), Her-2 (−), and TTF1 (−). These features suggested a metastatic adenocarcinoma of gingiva that was consistent with an origin in the patient’s gastric carcinoma. Then the patient received 3D–CRT (5000 cGy/25F/5w) with synchronization and sequential chemotherapy (raltitrexed 4 mg day1 q3w as 1 cycle). The gingival neoplasm disappeared, and serum level of CEA decreased to 3.31 ng/ml after radiotherapy and 4 cycles of chemotherapy. The disease was stable until 26 March 2017. And the follow-up is being performed.

## Discussion

Gastric carcinoma is one of the most prevalent malignancies in the world, especially in China. In spite of progress made in treatments over the past decade, distant metastasis is still the main cause of treatment failure, leading to poor prognosis in patients with gastric carcinoma. The most frequent sites of metastases from gastric carcinoma are lymph node, peritoneum, liver, lung, and ovaries. In addition, there have been some reports of unusual metastases in the uterus [4], testis [5], and oculus [6]. The process of gastric cancer metastasis is complicated, may include cancer cells to break through the basement membrane, adhesion, motor abilities, and anchoring to endothelium [7]. The major metastasis routes contain hematogenous channels, lymphatic vessels, the seeding of peritoneal surfaces, and direct extension. However, the detailed mechanism of gastric cancer metastasis is still not clear. Various cytokines and different signaling pathways change in the process of metastasis [8, 9]. These complex mechanisms can lead to some uncommon clinical presentations. For example, the incidence of gastric cancer metastasis to the oral cavity is extremely rare, especially to gingiva. To the best of our knowledge, only 18 cases, excluding this one, have been reported in English, Japanese, and Chinese literature to date (Table 1).

**Table 1:** Review of clinical and pathological data of gingival metastasis from gastric cancer

First author	Year	Sex/age	Site of gingiva mts	Time relation between primary tumor and mts	Histology	Other mts	Treatment	Follow-up
Lund [20]	1968	F/63	Mandibular	Synchronous	Undiff.	Yes	Surgery	Died, 2 weeks
Astacio [21]	1969	M/58	Mandibular	Synchronous	Poorly	ND	Chemotherapy	Died, 4 months
Ohba [22]	1974	M/51	Mandibular	Synchronous	Poorly	Yes	Radiotherapy	Died, 7 months
Lopez [23]	1976	F/65	Maxilla	3 weeks before primary	Poorly	Yes	None	Died, few days
Osaki [24]	1978	M/59	Maxilla	ND	Well	ND	Surgery	ND
Tojo [25]	1989	M/69	Mandibular	ND	Well	ND	Chemotherapy	Died, 5 months
Hamakawa [26]	1993	M/56	Mandibular	ND	Moderately	Yes	Surgery	Died, 4 months
Florio [27]	1995	M/66	Maxilla	3 months after primary	Moderately	No	Radiotherapy	Died, few weeks
Makino [28]	1997	M/60	Mandibular	ND	ND	ND	Chemotherapy	Died, 4 months
Yajima [29]	1999	M/65	Maxilla	ND	Well	No	Surgery	ND
Shimoyama [30]	2004	M/56	Mandibular	15 months after primary	Poorly	Yes	None	Died, 3 months
Colombo [17]	2005	F/61	Maxilla	7 months before primary	Undiff.	No	Radiochemo-therapy	Died, 15 months
Kwon [31]	2006	M/65	Mandibular	Synchronous	Poorly	No	None	Died, 3 months
Nishide [32]	2006	F/82	Mandibular	4 years after primary	Well	Yes	Surgery	ND
Hwang [33]	2007	M/58	Maxilla	4 years after primary	ND	Yes	Chemotherapy	ND
Sauerborn [16]	2011	M/70	Mandibular	3 months after primary	Moderately	Yes	Surgery	Died, 3 months
Guo [34]	2012	F/62	Mandibular	2 years after primary	Poorly	Yes	None	Died, 6 months
Kalaitsidou [35]	2015	M/71	Mandibular	2 years after primary	Poorly	ND	Surgery	ND
Current case	2017	M/75	Maxilla	2.5 years after primary	Poorly	Yes	Radiochemotherapy	Alive, in progress

*F* female, *M* male, *Mts* metastasis, *ND* not defined, *undiff* undifferentiated

Oral metastatic tumors comprise approximately 1% of malignant oral neoplasms, and most tumor lesions are in the jawbones with only a very small portion found in gingiva [10]. And once metastatic deposits in this organ, the location in gingiva is more common at the maxillary rather than at the mandibular gingival, and at the molar rather than at the anterior region [11]. The possible dissemination pathways of metastasis to the gingival are hematogenous channels, but the pathogenesis of the metastasis to the gingiva has been unclear so far. According to the comprehensive literature review, gingival metastatic tumor perhaps is connected to chronic periodontitis, which has been in existence before gingival metastasis. Bacterial pathogens stimulate the body’s inflammatory cells to produce reactive oxygen species (ROS) [12]. ROS are involved in the transcription and activation of a large series of cytokines and growth factors, which play a crucial role in the pathogenesis and the progression of cancer [13]. In this case, the patient presented with gingival swelling of durations about 2 months. In fact, his chronic periodontitis was present for several years.

Accurate diagnosis of gingival metastatic carcinoma depends on case history, clinical manifestation, imaging examination, and pathological detection. Significantly, pathologic evidence is considered as the most important one in diagnostic procedure. In this case, the similar cellular morphology and immunoreactivity of CK7, CK20, Villin, and MUC2 demonstrated the gingival metastasis originated from his gastric carcinoma. The upper gastrointestinal tract adenocarcinomas had been reported to be positive for both CK7 and CK20 in 78% of cases [14]. And MUC2 and Villin are also important gastric epithelial cell markers for phenotypical and histological detection of gastric cancer [15]. However, squamous carcinoma is the predominant type and negative for CK7, CK20, MUC2, and Villin in primary gingival cancer. Thus, the result of IHC is conducive to the diagnosis of metastatic tumors of gastric origin. Meanwhile, this case has some typical clinical features. Firstly, oral cavity metastasis from gastric cancer occurs primarily in elderly patients, and the average age was 63 years [16]. This patient is 75 years old, which is in accordance with the age distribution. Secondly, gingival metastatic carcinoma often rapidly progresses. The gingival mass of this case was rapidly enlarging within a month. Furthermore, the mass of gingiva in this case locates in the molar area of left maxillary, which is predilection site of gingival metastasis [17]. Finally, gingival metastasis usually accompanies by the metastasis of other organs at the same time [18]. The PET-CT result of this patient identified hypermetabolic lesions at the left adrenal gland, except for gingival, which are considered as metastatic lesions. In summary, the gingival metastasis is prone to misdiagnosis as epulis or periodontitis. In order to avoid misdiagnosis and the loss of the best treatment opportunities, such patients are expected to have a detailed oral and systemic check-up, a careful analysis of the medical history of malignancy, and definite pathological diagnosis.

Metastatic malignant tumors in the gingiva also exhibit an aggressive disease, which means that patients have been in advanced stage and have a poor prognosis [19]. The overall survival periods of such patients are no longer than a year. At present, extremely effective treatment is absent in line with the published literature. In the present case, swelling of gingiva causes disturbance of mastication and seriously affects the quality of life. Therefore, the aim of the treatment is to alleviate the symptoms and to improve the quality of life. This patient refused complete surgical intervention and then received the palliative radiotherapy for oral metastatic lesions and systemic intravenous chemotherapy. Taking the patient’s age, poorer tolerance, and first-line therapy failure into consideration, raltitrexed, a novel thymidylate synthase inhibitor, as single chemotherapeutic regimen is adopted. And the results of this paper show that, palliative combined radiochemotherapy is effective with mild toxicity. Furthermore, after reviewing all previous cases in Table 1, it is found that the patient of the longest survival also received radiochemotherapy. This probably means combined radiochemotherapy is a better selection for these special patients.

## Conclusions

In a word, this paper presents the clinicopathologic features of a gingival metastasis from gastric cancer found in a 75-year-old man. The particular pattern of spread in gastric cancer is rare, and the diagnosis is usually based on medical and exact pathological examination. Raltitrexed single regimen can be given with palliative radiotherapy, resulting in improved clinical benefit.

## Abbreviations

3D-CRT: Three-dimensional conformal radiotherapy
CEA: Carcinoembryonic antigen
CT: Computed tomography
IHC: Immunohistochemical
PET: Positron emission tomography
